# Variations of trophic structure and niche space in fish community along a highly regulated subtropical large river

**DOI:** 10.1002/ece3.9424

**Published:** 2022-10-17

**Authors:** Huijun Ru, Liqiao Zhong, Wei Nian, Yunfeng Li, Qiang Sheng, Zhaohui Ni

**Affiliations:** ^1^ Fishery Eco‐Environment Monitoring Center in Middle and Upper Reaches of Yangtze River Ministry of Agriculture and Rural Affairs of China/Yangtze River Fisheries Research Institute, Chinese Academy of Fisheries Science Wuhan China; ^2^ Zhejiang Province Key Laboratory of Aquatic Resources Conservation and Development, College of Life Sciences Huzhou University Huzhou China

**Keywords:** dam construction, energy flow, food webs, hydrogeomorphological condition, stable isotope niche

## Abstract

The trophic interactions between consumers and resources play a vital role in the stability of communities. In river systems, fragmentation of natural habitats and environmental changes alters the energy basis and community composition, consequently leading to variations in the community's trophic structure and niche space. However, our understanding of how the trophic structure responds to environmental changes is still very limited. Here, based on stable isotope data, we explored and compared trophic positions (TPs), community‐wide trophic metrics, and isotope niche space of fish communities in three reaches with different hydrogeomorphic conditions along a highly regulated subtropical river over three seasons. The community trophic structure and niche space showed notable spatiotemporal variations. Overall, the downstream reach had lower TPs, trophic diversity but higher trophic redundancy. The middle reach occupied a wider isotope niche space than other reaches, with the largest niche size during autumn. Furthermore, the niche overlap was relatively high in winter between reaches and in the downstream between seasons. The results implied a homogenization of feeding functional groups and energy flow pathways of species in the downstream community associated with the change of energy source and stability of hydrological conditions. The relationship between trophic structure and environmental factors suggested that the dam‐induced alteration in hydrological‐related aspects may drive the changes in the functional group composition, together with changes in energy basis, resulting in differences in the trophic structure of the community. The results of the present study deepen our understanding of how ecosystem functions respond to disturbance, thus contributing to improved ability to conserve river ecosystems.

## Graphical abstract

The trophic structure and niche overlap of fish communities exhibited notable spatiotemporal variability at the lower Jinsha River in reach‐scale. The fish communities in the reach downstream the dam had lower trophic position, trophic diversity but higher trophic redundancy and the niche overlap between seasons, implied the homogenization of feeding function group and energy flow pathways associated with the variations of the hydrogeomorphic condition.

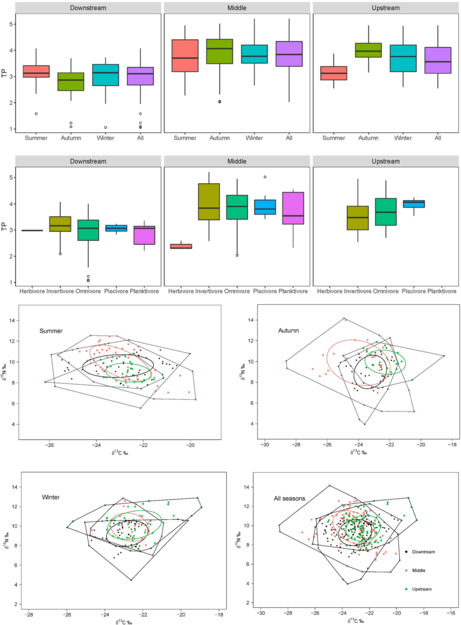

## 1 INTRODUCTION

The species composition and consumer‐resource interactions in the food web fundamentally govern how energy and materials flow in the ecosystem (Cross et al., 2013; Power et al., 1996) and thus play important roles in the stability of communities in a rapidly changing world (Dezerald et al., 2018). In natural river ecosystems, the properties of trophic structure in a community will vary spatially with respect to environmental differences associated with rainfall patterns, physiography, and total primary production (Winemiller, 1990), as well as temporally to a large extent with seasonal hydrological and (or) temperature variation (McMeans et al., 2019; Pease et al., 2019). In addition, emerging evidence indicates the vertical attribute of the trophic structure of community reflects flexible foraging behaviors in response to seasonal environmental fluctuations (McMeans et al., 2019). However, increasing human activities (e.g., dam building, intensive land use) are influencing the community trophic structure significantly (Hoeinghaus et al., 2007; Power & Dietrich, 2002; Price et al., 2019) and rapidly altering the spatial and temporal niches of animals (Manlick & Pauli, 2020). For example, fragmentation of river habitats alters ecological communities, causing the loss of co‐evolving trophic interacting species and introducing some new ones in the new community (Cross et al., 2013). Expected changes in food resources may be interrupted by flow alteration, resulting in discontinuities in spatial patterns of discharge and food resource availability within an ecosystem (East et al., 2017; Poff et al., 2007). Therefore, exploring the properties of trophic structures of ecological communities in an ecosystem where fundamental changes occurred will further deepen our understanding of how ecosystem function responds to disturbance.

As one of the major and forceful threats to river ecosystems worldwide, flow regulation by dams alters the flow regime (Lytle & Poff, 2004) and affects fish diversity and distribution (Cooper et al., 2016; Poff & Zimmerman, 2010), as well as energy source contributions (Pingram et al., 2014; Wang et al., 2014), thereby ultimately changing food web structure and ecosystem function (Cross et al., 2013; Mor et al., 2018). However, studies on the impacts of dams on fish communities have extensively focused on the evolution of community composition and evaluation methods so far (Cooper et al., 2016; Gao et al., 2019). Despite recent advances in different regions of the world, for example, the relationships between trophic ecology and environmental variability (Hur et al., 2018; Kaymak et al., 2018; Mazumder et al., 2016) and regional fishery alterations (Lima et al., 2020), our understanding of how the trophic structure responds to environmental changes is still very limited. Few studies have explored mechanistic links between variations of trophic structure in communities and its drivers, especially for subtropical regions where the natural flow regime has been substantially altered by dam construction (Grill et al., 2015).

Carbon and nitrogen stable isotope technology as a powerful tool has been widely used to explore the basal resource to consumers, diet tracing, and the energy flow in the food web (Duffill Telsnig et al., 2019; Layman et al., 2012; Nielsen et al., 2018; Zanden & Rasmussen, 2001). An increasing number of studies use isotopic data to extract quantitative information for trophic structure and niche analysis at the species or community level (Cucherousset & Villéger, 2015; Jackson et al., 2011; Layman, Arrington, et al., 2007a). As a proxy of ecological niches, the isotope niche metrics determined not only the space occupied by species packing within a food web in C‐N biplot but also relevant descriptor of the overall trophic status and resource use (Newsome et al., 2007), which were used to quantify the trophic diversity and niche overlap of coexisting species in the community (Abrantes et al., 2014; Masese et al., 2018). Furthermore, the community‐level metrics and the calculation of the standard ellipse area under the Bayesian framework make it easier to conduct comparative studies on community trophic structure across different temporal and spatial scales (Jackson et al., 2011).

Characterized by abundant water resources and rich biodiversity, the upper Yangtze River (known locally as the Jinsha River) has been marked as an ecological shelter and functional critical region of the Yangtze River (Sun, 2008). However, the ecological environment in this region is undergoing unprecedented changes due to extensive hydropower development (Chen et al., 2005). Regional fish habitat quality and species composition have been profoundly altered (He et al., 2010; Yang et al., 2014), and fish reproduction associated with water temperature has been significantly affected (Cheng et al., 2015; Luo et al., 2012). In a previous study, Ru et al. (2020) examined basal source and food web structure variation among different hydrogeomorphic river reaches in the lower Jinsha River. The results revealed that autochthonous sources were the main sources supporting most consumers. Their contributions increased gradually from upstream to downstream. At the same time, allochthonous sources (mainly C_3_ plants) played an important supplementary role in both reaches above the dam or the high flow period. Here, based on carbon (C) and nitrogen (N) stable isotopes data sets, we compared trophic structure and niche overlaps of fish communities in this highly regulated river at the reach scale over three seasons. We hypothesized that the differences in energy source basis and fish community structure, associated with flow regime alteration, would lead to variations in trophic structure and ecological niche attributes of the communities. Specifically, we predicted that compared to the upstream reach, the fish community downstream of the dam would show a compressed niche space and an increasing niche overlap between seasons as the reduced food resource diversity associated with dam‐induced hydrological stability (Ru et al., 2020). In contrast, greater trophic diversity and wider niche space would occur for the community in the middle reach (within the reservoir), where the food resources were much richer and a variety of functional groups with higher trophic positions inhabited (Wang et al., 2016). Then we also explored mechanistic links between community trophic metrics and environmental variables. We predicted that the factors affecting patterns of trophic structure would be hydrology‐related factors, which determined the river flow regime, controlling community composition, food resources, and ecological processes (Palmer & Ruhi, 2019). We expected our findings could improve our understanding of how community trophic structure responds to river damming and provide valuable insights into local fish conservation and fisheries management.

## 2 MATERIALS AND METHODS

### 2.1 Study area and sampling sites

The lower Jinsha River extends from the Yalong River confluence to Yibin city of Sichuan province, with a total length of 768 km and a drainage area of 214,000 km^2^. As it flows through the high mountain valley, the elevation changes up to 729 m, and the mean river gradient is about 0.93‰ (Xu et al., 2010). The river channel is constrained, and the riverbank slope is very steep. The vegetation along river banks is mostly sparse shrubs and herbs (Zheng et al., 2014). Influenced by the subtropical monsoon climate, the natural flow regime shows a strong seasonality, with a flood season usually from July to September (Cen et al., 2012). Like many subtropical rivers, precipitation is the main source of river runoff. Therefore, the river flow shows a good correlation with seasonal precipitation (Lu et al., 2016). As one of the richest regions of freshwater fish, a total of 56 endemic species in China were recorded and thus play a vital role in the protection and maintenance of rare and endemic fish (Jiang et al., 2009). However, with continuous economic development in this region, the river ecological environment has been increasingly disturbed by anthropogenic activities, particularly the dam construction. As yet four large hydropower stations have been planned and constructed in the lower Jinsha River. Consequently, the fish community structure has significantly altered, and many endemic fishes are being threatened, particularly in river segments directly below large dams (Chen et al., 2005; Gao et al., 2013).

**FIGURE 1:**
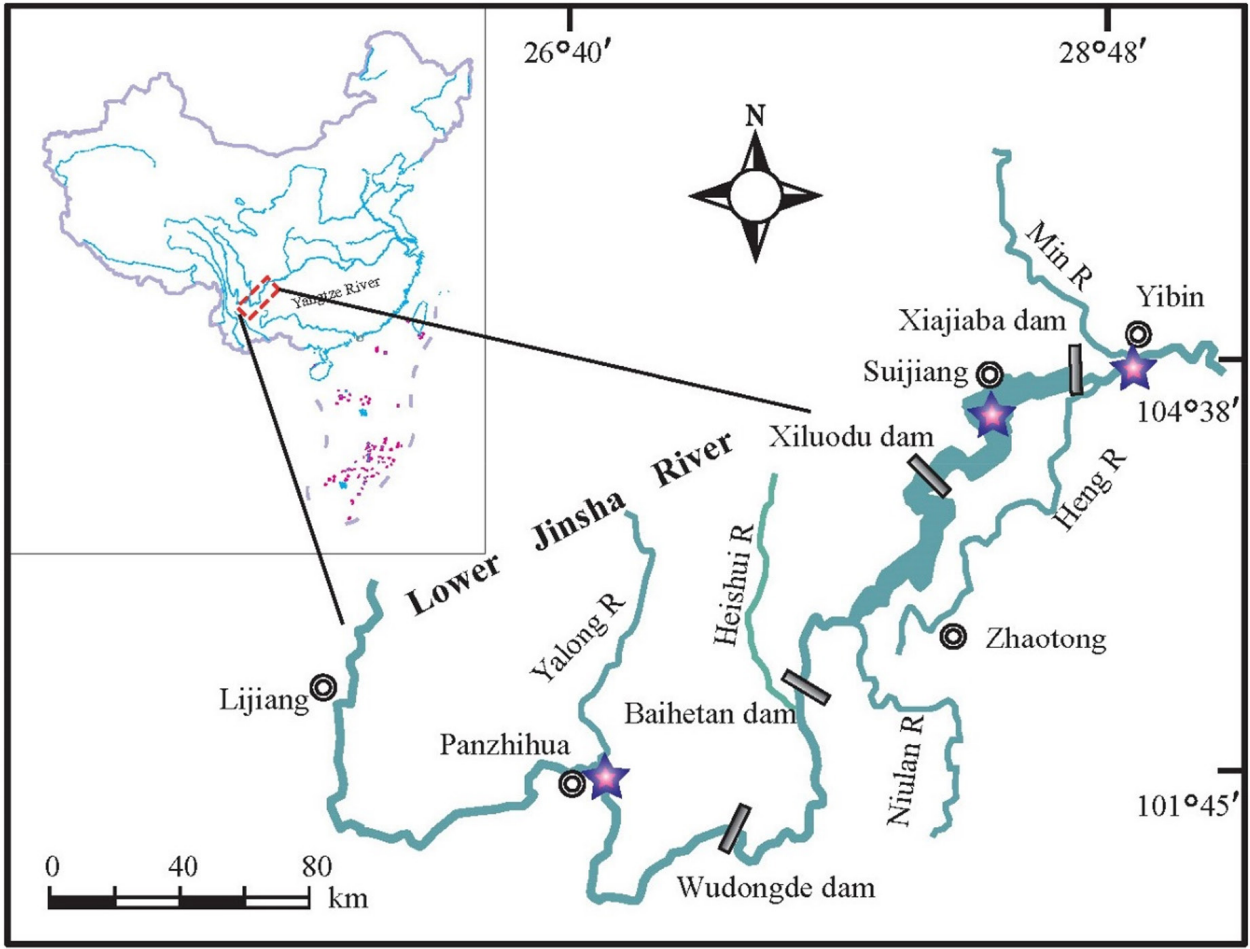
Map of the lower Jinsha River Basin, China, showing the locations of sampling sites, major tributaries and the dams.

This study was carried out at three reaches that differed in hydrogeomorphic conditions along the lower Jinsha River over three seasons (summer, autumn, and winter) in June, September, and December 2015. Since springtime is the forbidden period established by the Chinese government to protect fish reproduction, this season was not included in the study. Therefore, no sample collection was conducted during spring. The sampling site of the downstream reach was located near Baixi town, just 20 km downstream of the Xiangjiaba dam (Figure 1). In this section, riverbanks were not steep and with a relatively expansive terrace along both banks. Due to the dam regulation, the flow velocity decreased with relatively low turbidity. The hydrological process in this reach showed less hydrological variability in contrast to the upstream (Ru et al., 2020). In the middle reach, the sampling site was located ~52 km above the dam. Within the Xiangjiaba Reservoir, the river channel was much broader and deeper, with the mean width and depth of 1 km and 25 m, respectively. The water velocity at this site was much slower compared to the other two sites. For comparison purposes, we chose an upstream reach unaffected by the dams, so the flow regime remains in its natural state. The sampling site was located about 12 km downstream of Panzhihua City. Urbanization may have certain effects on this section's water quality and river biota (Yang & Dai, 2016). Excluding these factors, it is the ideal zone for our study. The river channel was narrower and more constrained, and the flow was characterized by high velocity and turbidity. In all three reaches, in‐stream macrophyte was absent. The riparian vegetation was grasses and shrubs, and the vegetation coverages tended to increase from upstream to downstream.

### 2.2 Environmental data and sample collection

The physical and chemical environmental variables were measured on the left, middle, and right of river cross‐section per reach and season synchronously. Water temperature (Temp.), conductivity (Cond.), dissolved oxygen (DO), pH, turbidity (Turb.), and Chlorophyll‐a were measured in situ by EXO2 Water Quality Sonde (YSI Inc., USA), water velocity was measured using velocity meters (FP 201; Global Water, USA). One liter of surface water sample was used to analyze for chemical oxygen demand (COD), total nitrogen (TN), total phosphorus (TP), and biological oxygen demand (BOD_5_) in the Laboratory of the Fishery Eco‐Environmental Monitoring Center of the Middle and Upper Reaches of the Yangtze River, Ministry of Agriculture and Rural Affairs, China.

The benthic macroinvertebrates were collected using a Peterson sampler in the middle channel and a Surber sampler or D‐shape net (300 μm mesh) along the littoral zone. To obtain a representative and sufficient sample of local fish assemblage, we used a combination of gillnets and fishing hooks in the main channel and minnow fishing bait traps around the littoral habitats at each sampling site. To conduct a unified sampling effort, we maintained identical amounts of nets and the same collection time at each sampling. Following the collection, all the samples were immediately placed in a portable icebox and then taken back to the laboratory for processing. In the laboratory, benthic macroinvertebrates were identified to order or family using keys provided by relevant literature (He, 1985; Morse et al., 1994). Each taxon was combined together at same reach during a sampling event and placed in distilled water for 24 h to empty their gut contents. To avoid the influence of carbonate on the δ^13^C ratios, shells of snail (*Radix* sp.) were removed and only the muscle parts remained. The 3–20 individuals of each species from the same sampling site were pooled as a single sample to ensure an adequate sample mass. Fish were identified to species, weighed to the nearest 0.1 g, measured standard length to 0.1 cm, and analyzed individually. Dorsal white muscle tissue samples were taken using a scalpel, rinsed with distilled water and inspected to ensure samples were free of bone, scales. All samples were dried in a constant temperature drying oven at 60°C for 48 h and then ground into a fine powder.

### 2.3 Stable isotope analyses

The stable carbon and nitrogen isotopes were analyzed using an isotope ratio mass spectrometer at the Stable Isotope Ratio Mass Spectrometry Laboratory, Chinese Academy of Forestry, Beijing, China. The standard material for carbon was Pee Dee Belemnite limestone, and the nitrogen standard was atmospheric nitrogen gas. The stable isotope results were expressed as parts per thousand (%) the deviations relative to the isotopic standards using the following formula:

$$\delta \;X=\left[\left(R_{\text{sample}}/R_{\text{standard}}\right)–1\right]\times 10^{3},$$

where *X* is the isotope ^13^C or ^15^N, and *R* is ^15^N/^14^N or ^13^C/^12^C. The measurement precision was approximately 0.1%and 0.3% for δ^13^C and δ^15^N, respectively.

### 2.4 Data analyses

We calculated fish trophic positions (TPs) using the following formula:

$$\mathrm{TPs}=2+\left(\delta^{15}N_{\text{cons}}–\delta^{15}N_{\text{base}}\right)/{\Delta \delta }^{15}N$$

where 2 is the TP of the invertebrate primary consumers used to estimate the baseline δ^15^N (δ^15^N_base_), δ^15^N_cons_ is the measured δ^15^N of the consumer for which TPs need to be estimated. The δ^15^N_base_ is the average δ^15^N of baseline consumers. We used benthic macroinvertebrate primary consumers, including oligochaetes, chironomids, and snails as the baseline, which can quantify the appropriate trophic position of secondary consumers in aquatic food webs (Post, 2002; Vander Zanden & Rasmussen, 1999). The trophic fractionation for δ^15^N (Δδ^15^N) can vary depending on the consumer's diet (McCutchan et al., 2003; Peterson & Fry, 1987). In our study, we divided fish into five feeding functional groups (Anonymous, 1976) and assigned 2.2% for herbivores and planktivores, 2.3% for omnivores and invertivores, and 3.3% for piscivores (McCutchan et al., 2003).

A two‐way ANOVA was used to compare environmental variables and TPs of fish communities among sites and seasons (Model: reach, season, reach × season). If the interaction effect of predictors was significant, the simple effect analysis of a single predictor was performed. The pairwise differences were tested using Tukey's HSD post hoc test. All the statistical analyses were performed using STATISTICA 10.0.

The trophic structure and niche space were described using five of Layman's community‐wide metrics (Layman, Arrington, et al., 2007a) and standard ellipse area (SEA) as proposed by Jackson et al. (2011) in each site and season. Stable isotope ratios of all fish species in each community were used to calculate the metrics. The five Layman's metrics were as follows: (a) δ^13^C range (CR), which depicts basal source diversity and breadth supporting consumers; (b) δ^15^N range (NR), which describes the trophic length; (c) the mean distance to centroid (CD), which is the mean Euclidean distance of each assemblage component to the centroid and a measure of community niche width (related to trophic diversity) and species spacing; (d) the mean nearest neighbor distance (MNND), which is the mean Euclidean distance from each group to its nearest neighbor in the δ^13^C–δ^15^N biplot space, an estimate of density and clustering of species within the community; and (e) the standard deviation of the nearest neighbor distance (SDNND), which measures the uniformity of the groups' spacing in the biplot space. Both MNND and SDNND provide information about trophic redundancy, whereby a small MNND means increased trophic redundancy, that is, there are many groups with similar trophic ecologies. A lower SDNND means more even species distribution, suggesting an increased trophic redundancy as different groups have more similar trophic ecologies (Layman, Arrington, et al., 2007a). The method of standard ellipse is comparable to the univariate SD and contains c. 40% of the data (Batschelet, 1981), which are unbiased concerning sample size, allowing us to make a robust comparison among data sets comprising different sample sizes. We presented the SEA results via Bayesian inference (Bayesian standard ellipse area, SEA_B_) and sample size‐corrected standard ellipse area (SEA_C_) based on Bessel's correction, which provides a highly satisfactory correction for all sample sizes. The degree of ellipse overlap was an indication of similarities or dissimilarities for food sources of consumers between pairs of sites or seasons (Abrantes et al., 2014; Masese et al., 2018); we also calculated the overlap of SEA_B_ and represented results by using 95% ellipse overlap in SEA_B_ between pairs of sites/seasons. All the calculations were performed using the SIBER package (Jackson et al., 2011; Parnell et al., 2010) in R.

To identify the effect of environmental variables on the feeding functional group and trophic structure, a two‐way hierarchical clustering analysis was performed by using the pheatmap package in R. Prior to analysis, Spearman’s correlation was performed on variables of two matrices firstly, and the correlation coefficient *ρ* was used to select uncorrelated variables and reduce the collinearity between continuous variables (Dormann et al., 2013). In this study, there are two pairs of highly correlated environmental variables, turbidity and DO, and COD_Mn_ and BOD_5_ (Spearman's *ρ >* 0.80), and two pairs of highly correlated trophic variables, SEA and CR, CD and CR; hence, we retained turbidity, conductivity, and CR in the later analysis. Cluster analysis grouped trophic variables by their correlation with environmental variables and enabled us to assess which specific environmental factors influenced these metrics. The results were displayed as a heatmap.

## 3 RESULTS

### 3.1 Environmental variables and functional community composition

The environmental variables varied in reach and season. The main effects of reach and season and their interaction effect were significant on all environmental variables except TP. There was no significant seasonal effect on TP. In each season, the simple effect of reach was also significant (Table S1). Water temperature, conductivity, and BOD_5_ were the highest in the middle and the lowest in the upstream reach, while turbidity and water velocity were the opposite. Chlorophyll‐a, COD_Mn_, TN, and TP were significantly lower in the middle reach than in other reaches (*p* < .005). DO and pH were significantly lower in the middle than in the upstream reach (*p* < .005). The substrate composition was mainly fine sand in the downstream reach, silt, and fine sand in the middle reach, sand, and gravel in the upstream reach (Table 1).

**TABLE 1:** Environmental variables and fish species number at each river reach in the lower Jinsha River during sampling periods.

	Water Temperature (°C)	Conductivity (μS/cm)	DO (μS/cm)	pH	Turbidity (FNU)	Chlorophyll‐a (μg/L)	COD_Mn_ (mg/L)	TN (mg/L)	TP (mg/L)	BOD_5_ (mg/L)	Water velocity (m/s)	Substrate type	Species number
Downstream												Sand	29
Summer	20.7	336.90	9.01	8.21	7.24	1.54	1.89	0.73	0.06	1.11	0.60		20
Autumn	21.4	307.10	10.28	8.48	44.65	0.15	1.79	1.60	0.14	1.02	0.80		12
Winter	19.0	260.40	8.92	8.48	4.60	0.30	0.52	0.91	0.08	0.62	0.55		17
Middle												Silt, gravel and sand	17
Summer	21.8	360.90	8.81	8.25	3.86	0.21	2.02	0.78	0.11	3.21	0.20		12
Autumn	21.4	276.70	9.74	8.35	24.79	0.19	1.51	0.81	0.05	1.09	0.32		12
Winter	18.2	272.65	9.30	8.50	4.44	0.27	0.65	1.19	0.07	0.69	0.25		8
Upstream												Sand and Gravel	18
Summer	19.6	327.38	9.51	8.13	10.40	0.54	1.82	1.20	0.09	1.36	1.00		10
Autumn	19.5	259.17	10.29	8.51	90.32	0.43	1.91	0.64	0.08	1.60	1.10		10
Winter	15.8	285.88	8.88	8.62	16.96	0.69	0.75	1.18	0.12	1.18	0.80		12

A total of 42 fish species were collected during the study. Among which, it was the highest in downstream, moderate in midstream, and the lowest in upstream. Including *Coreius guichenoti* and *Pseudobagrus crassilabris*, six fish species were collected in all sampling sites. Moreover, *Hypophthalmichthys molitrix* and *Hypophthalmichthys nobilis* were only collected in the middle reach (Table S2). The relative abundances and species richness of each feeding functional group differed among the river reaches. Overall, invertivores dominated upstream, while omnivores dominated in the middle and downstream. In contrast, species composition differed less between seasons at the same reach (Figure S1).

### 3.2 Fish stable isotope signatures and trophic positions

Fish δ^13^C and δ^15^N values broadly ranged from ‐ 30.8‰ to ‐ 12.1‰ and 4.0‰–14.2‰. The mean δ^13^C and δ^15^N values were −22.5 ± 1.3‰ (Mean ± SD), −23.2 ± 1.5‰, −22.8 ± 4.1‰ and 9.7 ± 1.3‰, 9.8 ± 1.5‰, 9.3 ± 1.4‰ in upstream, middle and downstream reach, respectively (Table S3). *Silurus meridionalis* collected in upstream reach and *Coreius heterokon* collected in middle reach had the highest and lowest δ^13^C values. *Culter alburnus* in middle reach and *Cyprinus carpio* in downstream reach had the highest and the lowest δ^15^N values.

**FIGURE 2:**
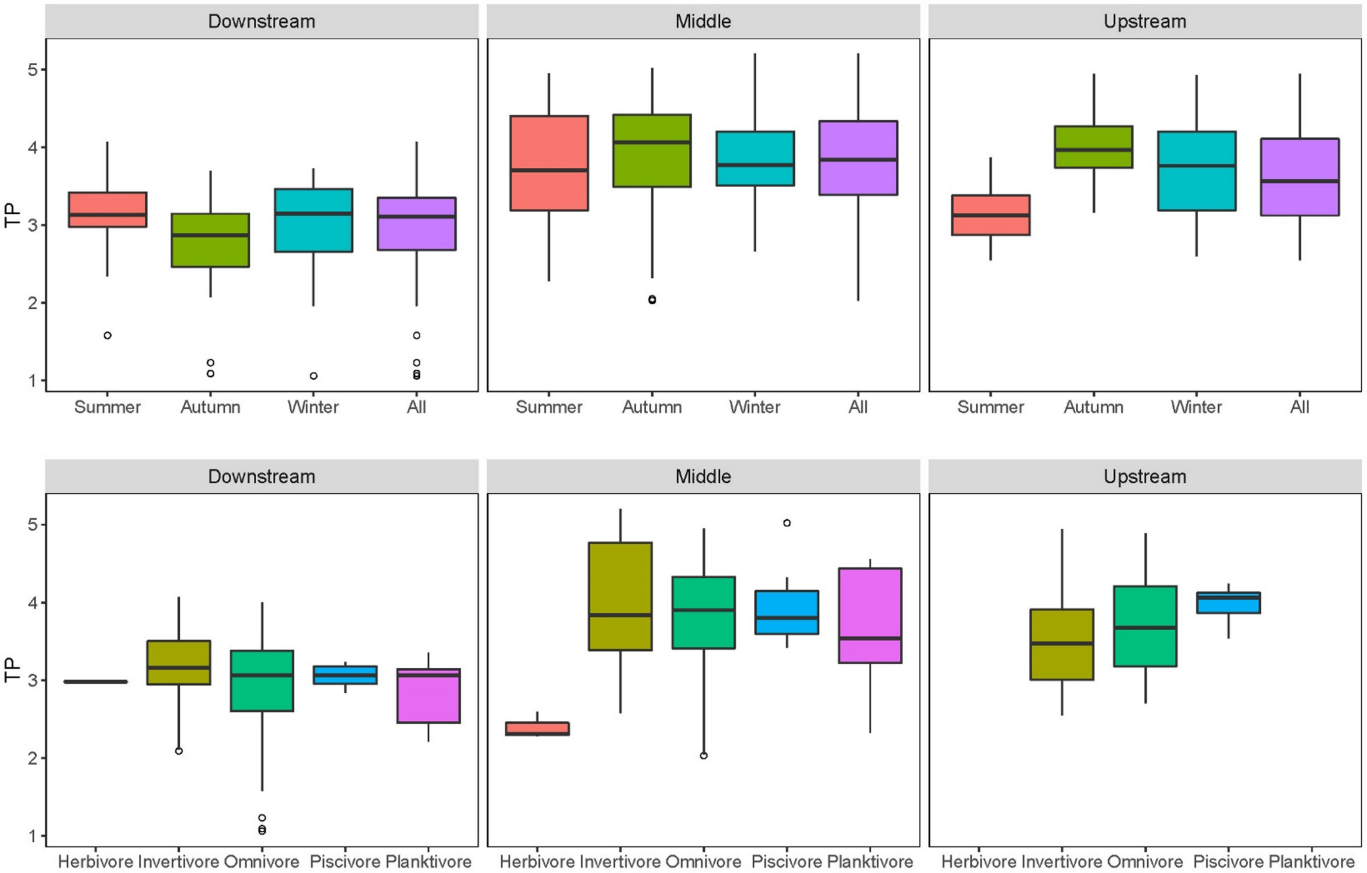
Trophic positions of fish communities at each river reach during summer, autumn, winter and all seasons (top) and different feeding groups (bottom) in the lower Jinsha River (Median; Box: 25–75%; Whisker: Min–Max excluding outliers, ○ = Outliers).

The TPs ranges were 2.6–4.9, 2.0–5.2, and 1.1–4.1 in the upstream, middle, and downstream reach. The effects of reaches, seasons, and their interaction on the TPs were significant (main effect, *F*_2,339_ = 60.21, *p* < .005; *F*_2,339_ = 5.53, *p* < .005; *F*_4,339_ = 12.07, *p <* .005), and the effects of reach in each season were also significant (simple effect, *F*_2,339_ = 15.69, *p* < .005; *F*_2,339_ = 43.44, *p* < .005; *F*_2,339_ = 23.43, *p* < .005). It was higher in winter and significantly lower in downstream (Tukey's post hoc test, *p <* .05) (Figure 2). All the invertivore, omnivore, and piscivore TPs show differences among reaches (one‐way ANOVA, *F*_2,113_ = 12.01, *p* < .001; *F*_2,176_ = 39.61, *p* < .001; *F*_2,113_ = 23.08, *p* < .001), and significantly lower in downstream (Tukey's post hoc test, *p <* .05). The planktivore TPs were significantly lower in downstream than in middle reach (Mann–Whitney *U*‐test, *U* = 20.00, *p* < .05).

### 3.3 Spatiotemporal differences in trophic structure and niche overlap

**FIGURE 3:**
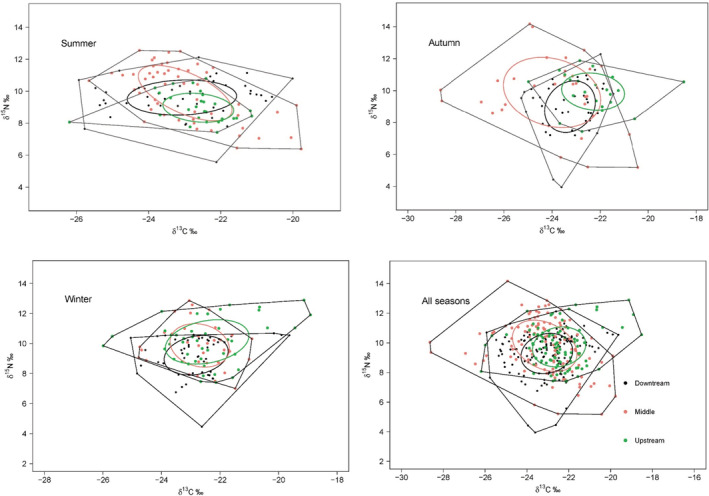
Isotopic spaces (based on δ^13^C ~ δ^15^N) occupied by fish communities at each river reach in the lower Jinsha River during summer, autumn, winter and all seasons combined. Solid line ellipses are the standard ellipse areas (SEA, containing 40% of the data) of fish communities at each reach during each season. Dotted lines are the convex hull areas of the fish communities.

**FIGURE 4:**
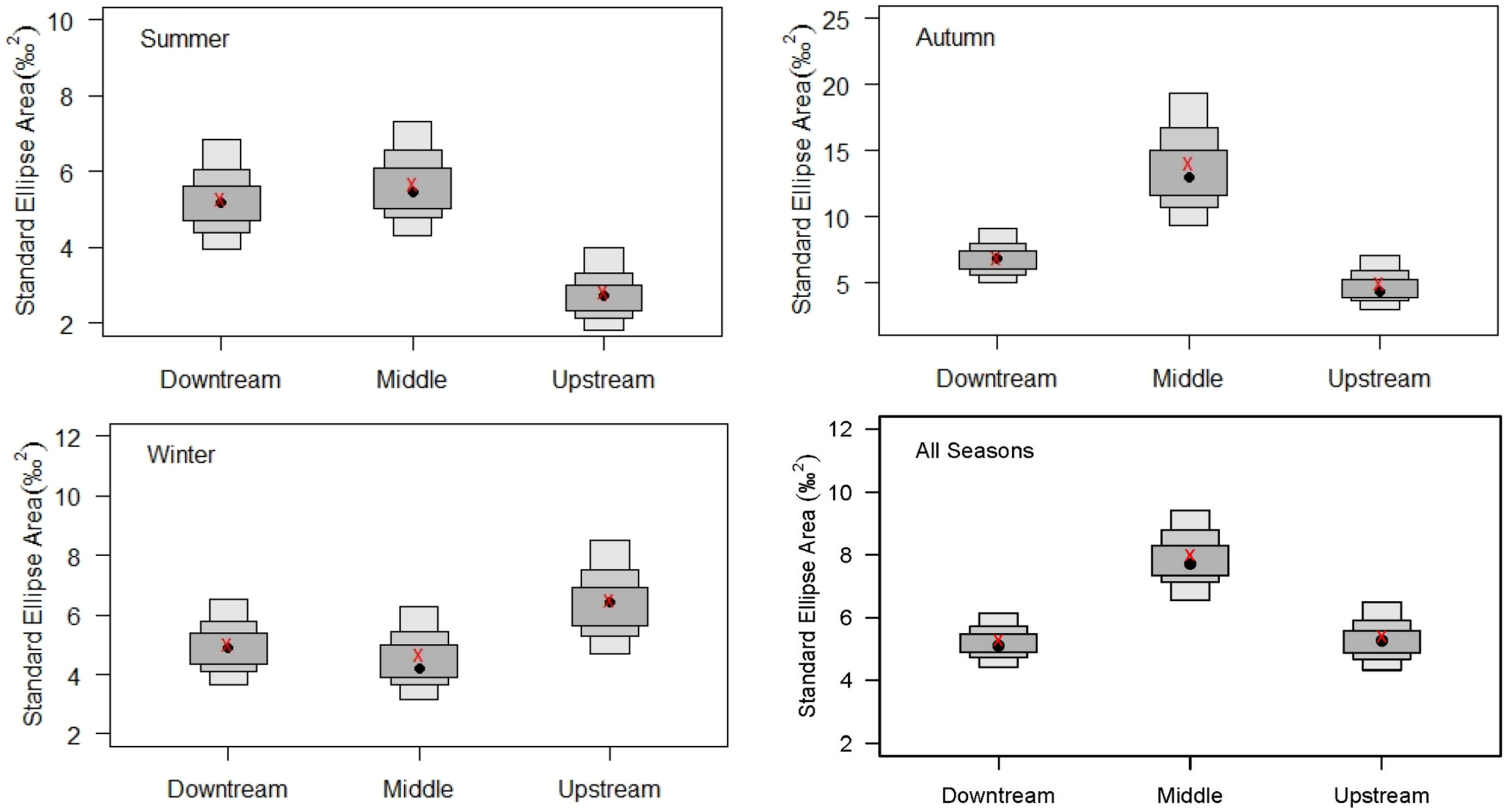
Density plots showing the credibility intervals of the standard ellipse areas (SEA). Black circles are the mode SEA, and boxes from wider to thinner indicate the 50%, 75% and 95% credible intervals for summer, autumn, winter and all seasons at each reach. Red crosses are the sample size‐corrected SEA (SEA_C_).

The fish isotopic spaces varied among reaches over time, which was reflected in the size and shape of SEA in the δ^13^C vs. δ^15^N biplots (Figure 3). Overall, the middle reach occupied a wider SEA than other reaches. The SEA was higher in autumn in the middle and downstream reaches, whereas in winter in the upstream. Similarly, SEA_B_ was the highest in the middle reach and the lowest in the upstream reach and decreased from upstream to downstream in winter (Figure 4 and Table 2). The SEA_B_ overlap between reaches was relatively high in winter, with the highest overlap (63.6%) between middle and upstream reach. It was lower in autumn and had the lowest overlap (25.2%) between the middle and upstream reach. The overlap between seasons was relatively higher (>50%) in downstream, while high overlap (68.7%) only occurred between summer and winter in the middle reach (Figure 4 and Table 2).

**TABLE 2:** Bayesian standard ellipse areas (SEA_B_) of fish community, 95% ellipse overlap between seasons for the same site and 95% ellipse overlap between pairs of sites for each season.

	SEA_B_ (‰^2^)	Proportion of 95% ellipse overlap between seasons (%)			Proportion of 95% ellipse overlap between pairs of sites (%)		
		Sum. vs. Aut.	Sum. vs. Win.	Aut. vs. Win.	Downstream	Middle	Upstream
Summer							
Downstream	4.7 (3.5–6.2)	54.7 (39.8–69.9)	67.2 (53.6–80.1)	59.7 (43.7–79.1)	100	51.0 (40.9–64.3)	55.7 (37.6–74.1)
Middle	5.7 (4.5–7.7)	42.3 (28.1–56.0)	68.7 (52.5–84.8)	33.0 (22.1–46.8)		100	43.9 (32.4–61.1)
Upstream	3.0 (2.1–4.5)	44.5 (31.4–60.4)	47.2 (30.6–63.1)	48.5 (31.3–67.2)			100
Autumn							
Downstream	7.4 (5.3–9.9)				100	48.3 (35.8–63.7)	39.4 (27.6–51.8)
Middle	12.0 (8.1–16.9)					100	25.2 (15.0–37.9)
Upstream	3.2 (1.9–4.9)						100
Winter							
Downstream	4.8 (3.7–6.4)				100	61.2 (50.4–78.4)	62.4 (51.5–75.1)
Middle	4.4 (3.2–6.3)					100	63.6 (46.5–81.5)
Upstream	6.0 (4.4–8.1)						100

*Note*: Overlaps (%) represent a proportion of the nonoverlapping areas of the two ellipses. Values are modes followed by 95% credibility intervals (in brackets).

**FIGURE 5:**
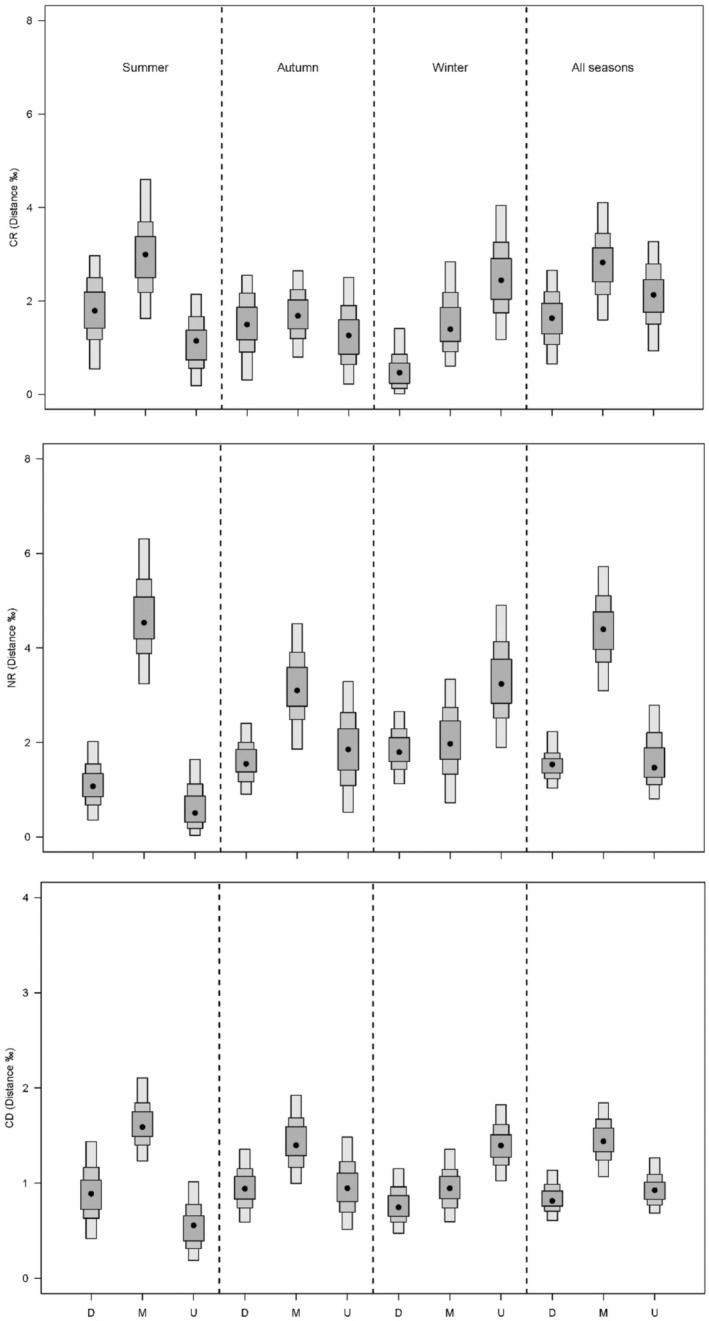
Layman community‐wide metrics (mode and 95% credible intervals) for trophic diversity information of the fish community at each reach and season: CR, range in stable isotope of carbon (δ^13^C); NR, range in stable nitrogen isotope δ^15^N; CD, mean distance to centroid.

**FIGURE 6:**
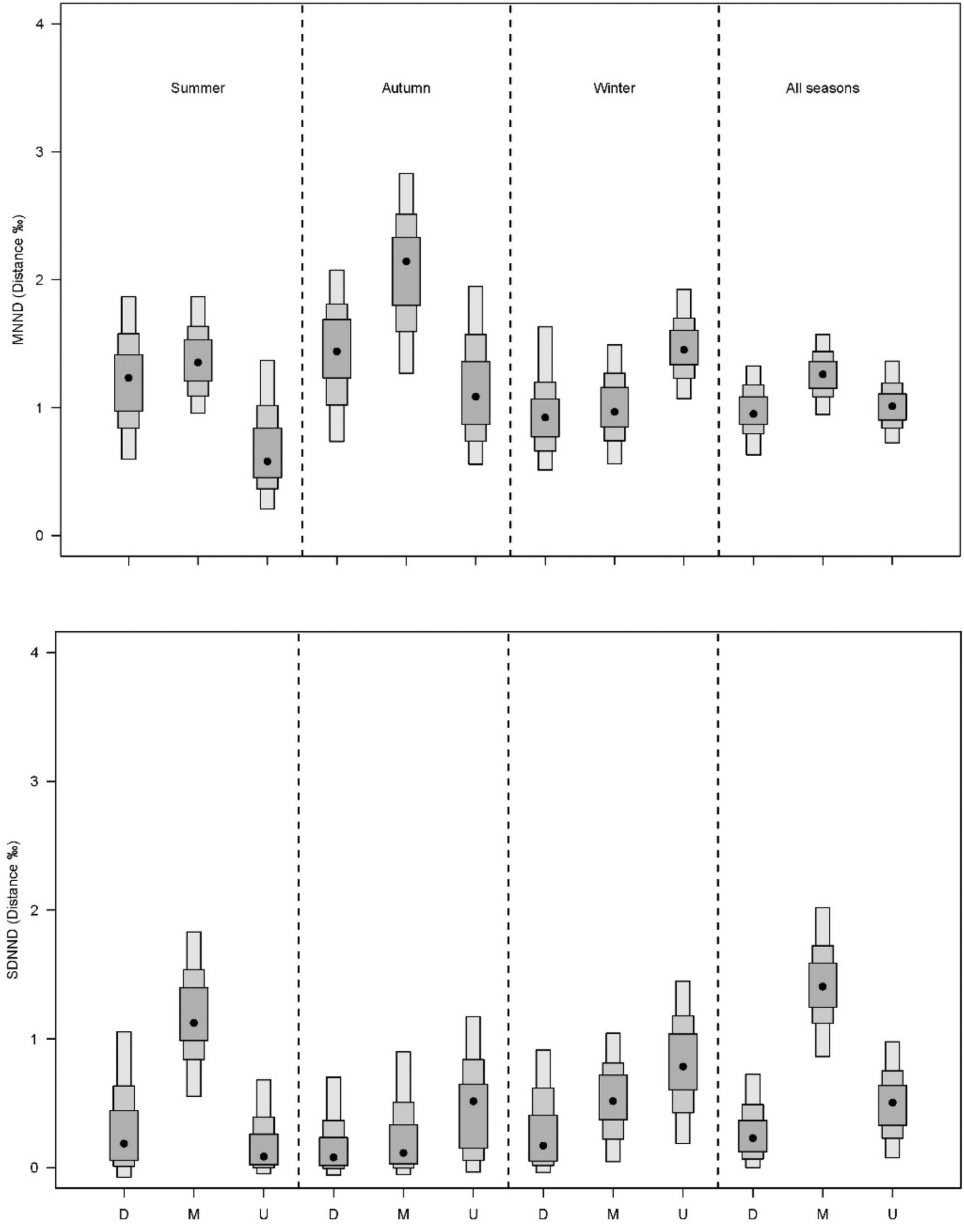
Layman community‐wide metrics (mode and 95% credible intervals) for trophic redundancy information of the fish community at each reach and season: MNND, mean nearest neighbour distance and SDNND, standard deviation of mean distance to centroid.

Overall, CR, CD, and NR were relatively high in the middle reach in summer and autumn while decreasing from upstream to downstream in winter. Regarding seasonal variations, these metrics varied at different degrees depending on river reach. It changed little in downstream, whereas relatively high in summer in the middle and in winter in the upstream (Figure 5). The MNND and SDNND were relatively low in downstream. Seasonal variations of both metrics were relatively small in downstream but more significant in the middle reach, where MNND was higher in autumn and SDNND in summer. In the upstream, they were higher in winter (Figure 6).

### 3.4 Associations among environmental variables and trophic structure measures

**FIGURE 7:**
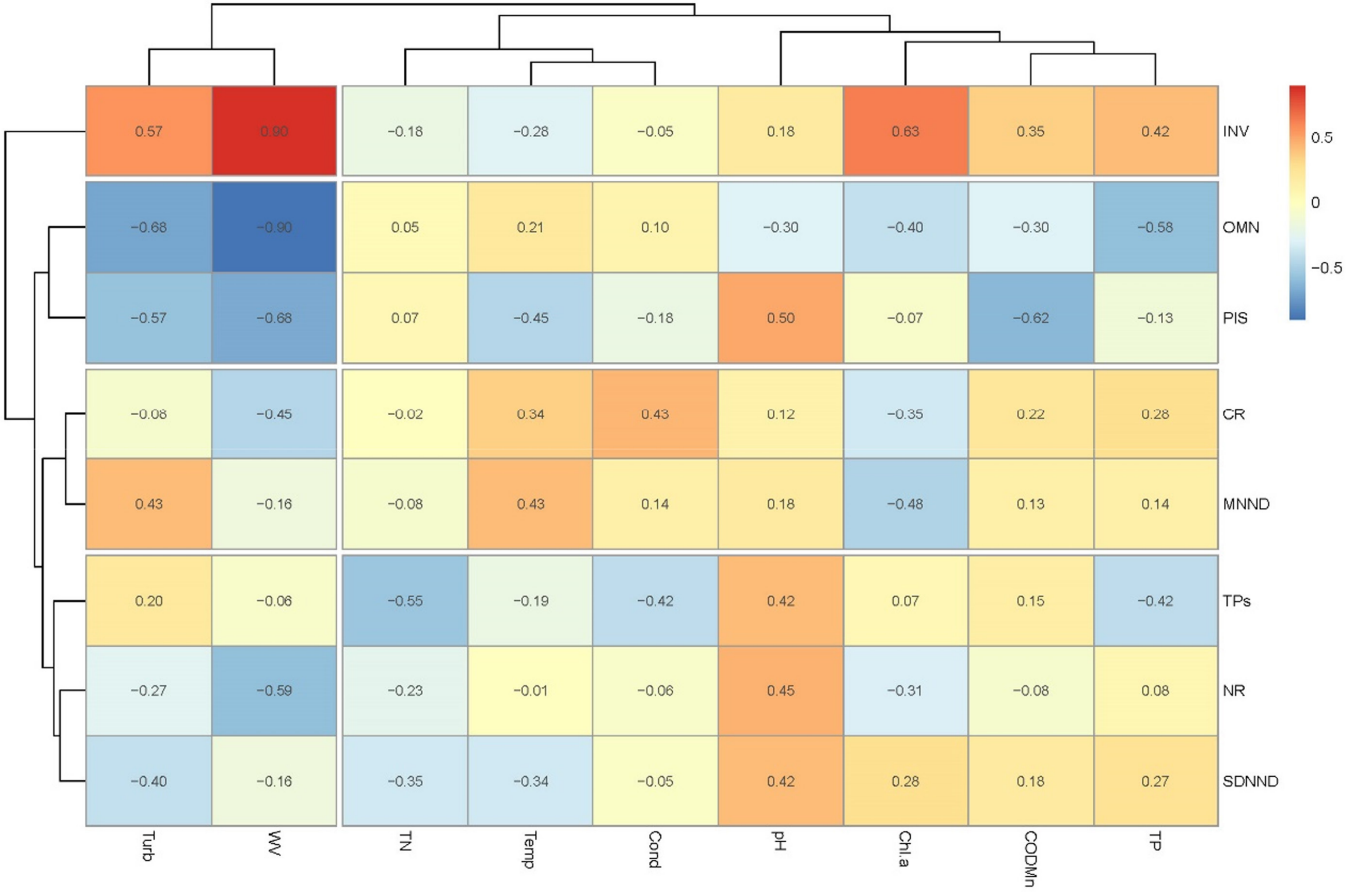
Heatmap of the correlations between the environmental variables and the trophic metrics. Similarities between trophic metrics are shown in the left clustering, while similarities between environmental variables are shown in the top clustering. Hierarchical clustering of samples was performed with Spearman correlation analysis and the average linkage algorithm.

The effects of the eight environmental variables on feeding functional groups and trophic metrics could be divided into two broad categories. Overall, water velocity and turbidity had high explanatory power on the proportions of feeding functional groups. Water velocity was significantly correlated with functional groups (*p* < .05). It was positively correlated with the abundance of invertivores while negatively with omnivores and piscivores. Turbidity was significantly negatively correlated with the abundance of omnivores (*p* < .05). Despite a high correlation between chlorophyll‐a and the invertivores, COD_Mn,_ and the piscivores, the relationships between most environmental variables and trophic metrics were not apparent (Figure 7).

## 4 DISCUSSION

### 4.1 Changes in functional community compositions and trophic positions

The ecological responses of fish communities to dam‐induced alterations in flow regime and habitat quality were complex (Delong & Thoms, 2016). In terms of the changes, the species composition of community change is more intuitive and obvious, which in turn has great potential effects on trophic complexity and niche breadth of the community. As a result, it ultimately affects the stability of the ecosystem (Mazumder et al., 2016; Mor et al., 2018; Ru et al., 2020). In this study, differences in functional group composition and TPs of the fish communities among the reaches revealed potential changes in the trophic structure at a reach scale in the lower Jinsha River system. Compared with pre‐dam construction (Liu et al., 2005), the abundance of bottom‐dwelling invertivore fish decreased either in the middle or downstream reach. In this regard, the results coincide with other surveys in this region. That is, the omnivores active in the upper and middle layers of water body significantly increased and had become the dominant species in this section (Li et al., 2013; Qu et al., 2020).

The trophic position estimation based on stable isotope provides a continuous measure of consumer trophic level, which integrates the assimilation of energy or mass flow through the different trophic paths leading to the consumers (Post, 2002). In our study, regardless of the whole community or various feeding functional groups, the TPs were lower in downstream and higher in middle reach during the sampling periods. On the one hand, it was likely related to available food resource types to primary consumers. In downstream, the primary consumers were mainly supported by autochthonous‐based sources, which had a higher δ^15^N value than allochthonous ones (Ru et al., 2020). As the baseline consumers had more enriched δ^15^N, consequently leading to a lower TP in fish. In addition, it was also related to the lower proportion of piscivores in this reach (7.1 ± 3.0%). The lack of large top predators, such as *Silurus meridionalis* and *Culter alburnus* that existed in the upstream and middle reach, also could lead to a decrease in TPs. The higher TPs in the middle reach should be closely related to dam‐induced nutrient retention and low water exchange, resulting in a high nutrient load in the reservoir environment (Wang et al., 2016). These factors would cause high δ^15^N in consumers that lived in a high nutrient load environment (Price et al., 2019). It is worth noting that extensive agricultural land use along the riparian area of the middle reach may also contribute to this. The high δ^15^N values of dissolved inorganic nitrogen from surrounding agricultural runoff would lead to high δ^15^N in fish (Winemiller et al., 2011).

### 4.2 Spatial differences in trophic structure and niche overlap

It is known that both natural and human‐caused disturbances will affect species distribution and the quantity and quality of available food resources along the longitudinal gradient of the river, which consequently leads to potential changes in the trophic structure of the community (Cross et al., 2013; Mor et al., 2018). Our results showed significant spatial differences in trophic structure and niche overlap among reaches along this highly regulated river. It is reasonable to infer that variations in the composition of functional groups and the energy basis of communities would contribute to these changes. As autochthonous source contribution increased while specialized feeding taxa reduced accordingly in downstream, the community's trophic diversity decreased. Despite the highest species richness, a significant proportion of species occupied similar niches (as showed lower MNND and SDNND). As a result, trophic niche space would be compressed with a concomitant increase in trophic redundancy.

In the middle reach, the highest Layman's metric values and SEA_B_ size indicated that food resources with different δ^13^C and the high community trophic diversity existed in such habitat. This finding coincided with research on rivers in other regions (Hoeinghaus et al., 2008; Kaymak et al., 2018). Generally, a community with a large isotope space may be composed of diverse trophic specialists (Bearhop et al., 2004) or have trophic generalists that could shift among alternative food sources (Layman, Quattrochi, et al., 2007b). There were also a proportion of planktivores and piscivores besides omnivores in the middle reach. Furthermore, the combination of high‐quality autochthonous sources with abundant allochthonous sources during high‐flow periods made a variety of food resources available for the community. In another study, Wang et al. (2016) also found that more prey taxa with higher trophic levels were available in reservoir habitats. In comparison, the upstream was furthest from the dam, and the trophic structure indicated that despite a low species richness, the fish community in the upstream utilized richer food resources and had relatively high trophic diversity (a medium CR and CD).

### 4.3 Seasonal differences in trophic structure and niche overlap

Our findings showed that the seasonal variations of the metrics and SEA_B_ overlaps differed depending on the reach, indicating there was a reach (or site)‐specific effect on the community trophic structure associated with seasonal hydrological and environmental conditions. Seasonal variabilities in hydrological and environmental factors caused temporal differences in habitat use and food resources availability, resulting in corresponding shifts in trophic interactions between consumer and resource in the community (Pease et al., 2019; Pool et al., 2017; Zheng et al., 2018). Compared to the middle and upstream reach, the metrics in downstream varied less seasonally, and the SEA_B_ overlapped greater between seasons (overlap >50%). It reflected an increase in similar resource utilization for species in the community. Evidence indicated that the dams hindered allochthonous matter inputs from upstream and riparian to downstream, and more stable hydrology conditions in the dam downstream promoted autochthonous production increasing (Ru et al., 2020). In turn, this also implied the impacts of river fragmentation on the feeding functional groups in the community and the homogenization of the energy flow pathways to predators (Layman, Quattrochi, et al., 2007b). In contrast, it varied significantly in the middle reach between the seasons, suggesting a variable habitat and resource availability. The summer's highest CR, NR, and CD (representing the trophic diversity) was likely related to higher primary productivity in the water, which arises from a higher solar irradiance and temperature in reservoir habitats (Kaymak et al., 2018). The larger niche space and low seasonal overlap in autumn reflected a dynamic response of species to changes in food resources or habitat under the condition of high flow and turbidity, which may suggest that there were more sizeable interspecific variations in the community. With the increasing seasonal hydrological variability in the upstream reach, the variations of the trophic metrics and niche space were more pronounced. The lowest seasonal SEA_B_ overlaps (44.5–48.5%) also implied that the fish consumed multiple food sources with different δ^13^C over time, which could be demonstrated by the diversity of underlying energy sources in this reach (Ru et al., 2020). Restricted by the fishing ban, we were unable to collect samples in the spring and thus could not evaluate the seasonal variations within a whole hydrological cycle throughout the year. Nevertheless, according to the hydrological process of the river, the hydrological characteristics were relatively similar in spring and winter (Ru et al., 2020). The results presented here may allow us to have a comparatively comprehensive understanding of the seasonal variations of the trophic structure of fish communities.

### 4.4 Relationships between community trophic structure and environmental variable

Previous studies showed that the flow regime alteration affected the composition of feeding groups and source basis by changing the primary productivity and subsidies between habitats (Cross et al., 2013; Zeug & Winemiller, 2008), resulting in significant changes in consumer‐resource trophic interactions and energy flow paths in the ecosystem (Turner et al., 2015). In this study, we tried to explore mechanism links between the trophic structure metrics and environmental factors. We found that among all the selected environmental variables, the hydrology‐related variables, such as water velocity and turbidity, were significantly correlated to the abundance of several functional groups. In contrast, other chemical water quality variables were not significantly correlated to various trophic metrics. These findings may suggest the alteration of flow regime associated with hydrological factors drove changes in the composition of functional groups, together with changes in energy basis, resulting in differences in the trophic structure pattern. Additionally, regarding the processes of trophic structure reshaping in the fish community, water chemical quality variables likely did not play the role directly. After the dam’s construction in the lower Jinsha River Basin, it was evident that the river reaches differently affected by the dam showing significant differences in hydrological characteristics (Qu et al., 2020). The essential hydrological elements for the growth and reproduction of lotic fish species, for example, *Coreius guichenoti*, have disappeared in the most dam‐affected reaches. The stability of the population has been threatened (Li et al., 2020). Regional productivity and energy bases supported consumers have changed (Ru et al., 2020). Given a short period covered by our study, it must be acknowledged that this also limited our ability to establish more robust relationships between trophic metrics and environmental factors. However, these findings highlighted that the research on the isotope‐based trophic structure of the community could provide complementary pathways to deepen our understanding of the impacts of increasing human activities on the river ecosystem beyond the physicochemical environment changes of water.

## 5 CONCLUSION

Exploring the trophic structure and niche of ecological communities in rapidly changing environments will contribute to our understanding of how ecosystem functions respond to disturbance. Our results demonstrated notable spatiotemporal variations of trophic structure and niche space in fish communities along a highly regulated subtropical river among reaches in response to different hydrogeomorphic conditions. The fish community downstream of the dam had a low trophic position and trophic diversity and compressed trophic niche space, implying a homogenization in energy flow pathways in the community. The results further showed that dam‐induced hydrological alteration, which may drive changes in the composition of functional groups together with changes in energy basis, resulted in differences in the trophic structure pattern. More research is needed to explore how the trophic structures of fish species with different feeding strategies or feeding functional groups respond to hydrological alteration in the future. Undoubtedly, it is critical to understand how community trophic function and food web stability respond to these changes and thus are helpful in mitigating the impact of dams on the function of river systems.

## AUTHOR CONTRIBUTIONS

**Hui‐Jun Ru:** Conceptualization (lead); data curation (lead); formal analysis (equal); funding acquisition (equal); investigation (lead); methodology (equal); project administration (equal); writing – original draft (lead); writing – review and editing (lead). **Liqiao Zhong:** Conceptualization (equal); data curation (equal); formal analysis (equal); methodology (equal); writing – review and editing (equal). **Nian Wei:** Formal analysis (equal); methodology (equal); resources (supporting); writing – review and editing (supporting). **Yunfeng Li:** Funding acquisition (equal); project administration (equal); resources (supporting). **Qiang Sheng:** Investigation (equal); methodology (supporting); software (supporting). **Zhaohui Ni:** Conceptualization (equal); writing – original draft (supporting); writing – review and editing (supporting).

## CONFLICT OF INTEREST

The authors of this manuscript have no conflict of interest to declare.

## Supporting information


Appendix S1

## Data Availability

The data that support the findings of this study are openly available in the Dryad Digital Repository at https://doi.org/10.5061/dryad.51c59zw7z.
