# Supplementary material for: Variations of trophic structure and niche space in fish community along a highly regulated subtropical large river
Source: Ecol Evol. 2022 Oct 17;12(10):e9424. doi: 10.1002/ece3.9424 (PMC9596329; doi:10.1002/ece3.9424)
Supplement: Supplementary file 1 — Appendix S1 [file ECE3-12-e9424-s001.docx]

Table S1 Two-way ANOVA of environmental variables among reaches, seasons and their interaction.

|  | Main effect and interaction effect | |  |  |  |  | Reach simple effect | |  |  |  |  |
| --- | --- | --- | --- | --- | --- | --- | --- | --- | --- | --- | --- | --- |
|  | Efffect | SS | Degr. Of Freedom | MS | F | *p* | Efffect | SS | Degr. Of Freedom | MS | F | *p* |
| Temp | reach | 26.42 | 2 | 13.21 | 3903.24 | < 0.005 | Summer | 5.66 | 2 | 2.83 | 836.25 | < 0.005 |
|  | season | 56.50 | 2 | 28.25 | 8347.75 | < 0.005 | Autumn | 7.00 | 2 | 3.50 | 1034.39 | < 0.005 |
|  | reach * season | 4.53 | 4 | 1.13 | 334.65 | < 0.005 | Winter | 18.29 | 2 | 9.14 | 2701.91 | < 0.005 |
| Cond | reach | 1217.61 | 2 | 608.80 | 25.90 | < 0.005 | Summer | 1627.01 | 2 | 813.50 | 34.61 | < 0.005 |
|  | season | 28361.80 | 2 | 14180.90 | 603.25 | < 0.005 | Autumn | 3529.18 | 2 | 1764.59 | 75.07 | < 0.005 |
|  | reach * season | 4322.08 | 4 | 1080.52 | 45.97 | < 0.005 | Winter | 383.50 | 2 | 191.75 | 8.16 | < 0.005 |
| DO | reach | 0.47 | 2 | 0.23 | 31.50 | < 0.005 | Summer | 1.02 | 2 | 0.51 | 68.56 | < 0.005 |
|  | season | 6.99 | 2 | 3.49 | 470.81 | < 0.005 | Autumn | 0.60 | 2 | 0.30 | 40.30 | < 0.005 |
|  | reach * season | 1.47 | 4 | 0.37 | 49.42 | < 0.005 | Winter | 0.32 | 2 | 0.16 | 21.48 | < 0.005 |
| pH | reach | 0.03 | 2 | 0.02 | 14.67 | < 0.005 | Summer | 0.05 | 2 | 0.02 | 3.47 | < 0.05 |
|  | season | 0.60 | 2 | 0.30 | 287.03 | < 0.005 | Autumn | 0.01 | 2 | 0.00 | 21.26 | < 0.005 |
|  | reach * season | 0.07 | 4 | 0.02 | 16.43 | < 0.005 | Winter | 0.04 | 2 | 0.02 | 22.80 | < 0.005 |
| Turb | reach | 3635.17 | 2 | 1817.59 | 722.10 | < 0.005 | Summer | 56.86 | 2 | 28.43 | 11.30 | < 0.005 |
|  | season | 12470.26 | 2 | 6235.13 | 2477.12 | < 0.005 | Autumn | 6776.00 | 2 | 3388.00 | 1346.00 | < 0.005 |
|  | reach * season | 3433.84 | 4 | 858.46 | 341.05 | < 0.005 | Winter | 236.14 | 2 | 118.07 | 46.91 | < 0.005 |
| Chl-a | reach | 0.98 | 2 | 0.49 | 93.43 | < 0.005 | Summer | 3.06 | 2 | 1.53 | 290.41 | < 0.005 |
|  | season | 1.32 | 2 | 0.66 | 125.31 | < 0.005 | Autumn | 0.13 | 2 | 0.07 | 12.62 | < 0.005 |
|  | reach * season | 2.34 | 4 | 0.59 | 111.21 | < 0.005 | Winter | 0.14 | 2 | 0.07 | 12.81 | < 0.005 |
| CODMn | reach | 0.06 | 2 | 0.03 | 54.88 | < 0.005 | Summer | 0.06 | 2 | 0.03 | 60.25 | < 0.005 |
|  | season | 8.54 | 2 | 4.27 | 8362.81 | < 0.005 | Autumn | 0.25 | 2 | 0.12 | 242.98 | < 0.005 |
|  | reach * season | 0.33 | 4 | 0.08 | 162.67 | < 0.005 | Winter | 0.08 | 2 | 0.04 | 76.99 | < 0.005 |
| TN | reach | 0.11 | 2 | 0.05 | 8.73 | < 0.005 | Summer | 0.42 | 2 | 0.21 | 25.98 | < 0.005 |
|  | season | 0.16 | 2 | 0.08 | 13.15 | < 0.005 | Autumn | 1.58 | 2 | 0.79 | 51.39 | < 0.005 |
|  | reach * season | 2.05 | 4 | 0.51 | 85.03 | < 0.005 | Winter | 0.15 | 2 | 0.08 | 105.83 | < 0.005 |
| TP | reach | 0.00 | 2 | 0.00 | 23.72 | < 0.005 | Summer | 0.004 | 2 | 0.00 | 62.77 | < 0.005 |
|  | season | 0.00 | 2 | 0.00 | 0.57 | 0.573 | Autumn | 0.01 | 2 | 0.01 | 176.60 | < 0.005 |
|  | reach * season | 0.02 | 4 | 0.01 | 144.38 | < 0.005 | Winter | 0.01 | 2 | 0.00 | 73.12 | < 0.005 |
| BOD5 | reach | 2.52 | 2 | 1.26 | 5224.15 | < 0.005 | Summer | 7.91 | 2 | 3.96 | 1643.68 | < 0.005 |
|  | season | 6.69 | 2 | 3.34 | 13889.87 | < 0.005 | Autumn | 0.59 | 2 | 0.30 | 1232.77 | < 0.005 |
|  | reach * season | 6.01 | 4 | 1.50 | 6240.65 | < 0.005 | Winter | 0.02 | 2 | 0.01 | 37.99 | < 0.005 |
| WV | reach | 2.27 | 2 | 1.14 | 581.88 | < 0.005 | Summer | 0.94 | 2 | 0.47 | 241.84 | < 0.005 |
|  | season | 0.20 | 2 | 0.10 | 51.90 | < 0.005 | Autumn | 0.93 | 2 | 0.47 | 238.24 | < 0.005 |
|  | reach * season | 0.06 | 4 | 0.02 | 8.15 | < 0.005 | Winter | 0.46 | 2 | 0.23 | 118.11 | < 0.005 |

Table s2 Fish species and their feeding functional groups at each reach in the lower Jinsha river. EF, denotes the endemic fish species of the Yangtze River Basin.

| Family | Fish species | Downstream reach | Middle reach | Upstream reach | Trophic guild | EF |
| --- | --- | --- | --- | --- | --- | --- |
| Cyprinidae | *Opsariichthys bidens* |  | + |  | Omnivore |  |
| Cyprinidae | *Zacco platypus* |  | + |  | Omnivore |  |
| Cyprinidae | *Hemiculter leucisculus* | + | + |  | Planktivore |  |
| Cyprinidae | *Pseudolaubuca sinensis* | + |  |  | Planktivore |  |
| Cyprinidae | *Culter alburnus* |  | + |  | Piscivore |  |
| Cyprinidae | *Mylopharyngodon piceus* |  | + |  | Invertivore |  |
| Cyprinidae | *Ctenopharyngodon idellus* |  | + |  | Herbivore |  |
| Cyprinidae | *Megalobrama amblycephala* | + |  |  | Herbivore |  |
| Cyprinidae | *Hypophthalmichthys molitrix* |  | + |  | Planktivore |  |
| Cyprinidae | *Hypophthalmichthys nobilis* |  | + |  | Planktivore |  |
| Cyprinidae | *Hemibarbus labeo* | + |  |  | Omnivore |  |
| Cyprinidae | *Hemibarbus maculatus* | + |  |  | Omnivore |  |
| Cyprinidae | *Saurogobio dabryi* | + | + |  | Omnivore |  |
| Cyprinidae | *Squalidus argentatus* | + |  |  | Omnivore |  |
| Cyprinidae | *Rhinogobio typus* | + |  |  | Invertivore |  |
| Cyprinidae | *Rhinogobio ventralis* | + |  | + | Invertivore | Y |
| Cyprinidae | *Coreius guichenoti* | + | + | + | Omnivore | Y |
| Cyprinidae | *Coreius heterokon* | + | + |  | Omnivore |  |
| Cyprinidae | *Cyprinus carpio* | + | + | + | Omnivore |  |
| Cyprinidae | *Carassius auratus* | + | + | + | Omnivore |  |
| Cyprinidae | *Garra pingi* | + |  |  | Omnivore |  |
| Cyprinidae | *Procypris rabaudi* | + |  |  | Omnivore | Y |
| Cyprinidae | *Gobiobotia filifer* | + |  |  | Invertivore |  |
| Cyprinidae | *Xenophysogobio boulengeri* | + |  |  | Invertivore | Y |
| Cyprinidae | *Xenophysogobio nudicorpa* |  |  | + | Invertivore | Y |
| Balitoridae | *Lepturichthys fimbriata* | + |  | + | Omnivore |  |
| Balitoridae | *Jinshaia sinensis* | + |  | + | Invertivore | Y |
| Balitoridae | *Jinshaia abbreviata* | + |  |  | Invertivore | Y |
| Cobitidae | *Botia superciliaris* | + |  | + | Invertivore |  |
| Cobitidae | *Leptobotia elongata* | + |  | + | Invertivore | Y |
| Cobitidae | *Leptobotia microphthalrna* | + |  |  | Omnivore | Y |
| Siluridae | *Silurus asotus* | + | + | + | Piscivore |  |
| Siluridae | *Silurus meridionalis* |  |  | + | Piscivore |  |
| Sisoridae | *Glyptothorax sinense* | + |  | + | Invertivore |  |
| Bagridae | *Pseudobagrus crassilabris* | + | + | + | Invertivore |  |
| Bagridae | *Pseudobagrus emarginatus* |  | + | + | Invertivore |  |
| Bagridae | *Leiocassis longirostris* | + |  |  | Piscivore |  |
| Bagridae | *Tachysurus nitidus* |  | + |  | Omnivore |  |
| Bagridae | *Tachysurus fulvidraco* | + |  |  | Omnivore |  |
| Bagridae | *Pseudobagrus vachellii* | + | + | + | Omnivore |  |
| Amblycipitidae | *Liobagrus marginatus* |  |  | + | Invertivore |  |

Table s3 Mean values (± SD) of **δ**^13^C, **δ**^15^N, body size and trophic levels of fish species in the community at each reach in the lower Jinsha river.

| Fish species | N | δ**^13^C** | δ**^15^N** | Body length (cm) | Body Weight (g) | Trophic level |
| --- | --- | --- | --- | --- | --- | --- |
| **Upstream reach** |  |  |  |  |  |  |
| *Rhinogobio ventralis* | 1 | -23.3 | 10.5 | 16.7 | 73.3 | 3.9 |
| *Coreius guichenoti* | 18 | -22.9±1.5 | 10.2±1.1 | 22.7±6.2 | 254.5±182.9 | 4.0±0.6 |
| *Cyprinus carpio* | 1 | -21.9 | 8.8 | 24.3 | 480.8 | 3.7 |
| *Carassius auratus* | 5 | -22.6±0.9 | 7.9±0.3 | 14.4±5.1 | 125.0±152.7 | 3.1±0.3 |
| *Xenophysogobio nudicorpa* | 2 | -23.1±0.6 | 8.2±1.1 | 7.8±1.1 | 7.9±2.5 | 2.8±0.3 |
| *Leptobotia elongata* | 6 | -22.4±0.4 | 10.3±0.3 | 20.4±7.7 | 147.8±161.9 | 3.5±0.2 |
| *Lepturichthys fimbriata* | 3 | -25.0±1.5 | 9.7±0.9 | 13.3±1.6 | 15.9±7.4 | 3.8±0.4 |
| *Jinshaia sinensis* | 3 | -22.8±0.3 | 8.9±0.4 | 10.9±1.6 | 20.4±6.7 | 3.0±0.1 |
| *Botia superciliaris* | 1 | -22.8 | 11.9 | 13.1 | 28.9 | 4.2 |
| *Silurus asotus* | 2 | -22.2±0.3 | 11.5±1 | 19.8±2.6 | 78.9±46.7 | 3.8±0.4 |
| *Silurus meridionalis* | 5 | -20.5±1.7 | 12.1±0.9 | 27.0±7.6 | 203.6±162.9 | 4.0±0.2 |
| *Glyptothorax sinense* | 7 | -23.1±0.4 | 10.1±1 | 8.6±0.7 | 13.1±2.5 | 3.5±0.4 |
| *Pseudobagrus crassilabris* | 23 | -21.9±1.1 | 9.4±1.4 | 14.0±4.3 | 39.8±31.1 | 3.7±0.7 |
| *Pseudobagrus emarginatus* | 2 | -22.1±0.8 | 10.4±0.7 | 10.1±1.6 | 18.3±5.1 | 4.4±0.3 |
| *Pseudobagrus vachelli* | 2 | -22.1±1.4 | 9.4±1.1 | 15.2±2.7 | 69.5±36.6 | 3.8±0.5 |
| *Tachysurus fulvidraco* | 5 | -22.3±0.4 | 8.7±0.5 | 11.7±2.0 | 31.8±12.8 | 3.1±0.2 |
| *Liobagrus marginatus* | 7 | -22.8±0.6 | 9.2±0.8 | 10.3±1.1 | 19.7±4.4 | 3.1±0.3 |
| **Middle reach** |  |  |  |  |  |  |
| *Opsariichthys bidens* | 1 | -23.5 | 12.1 | 13.6 | 40.0 | 4.9 |
| *Zacco platypus* | 1 | -25.0 | 11.1 | 13.5 | 64.0 | 4.3 |
| *Hemiculter leucisculus* | 4 | -23.7±0.9 | 9.2±1.5 | 15.4±1.2 | 42.3±12.4 | 3.7±0.7 |
| *Culter alburnus* | 1 | -24.9 | 14.2 | 18.3 | 84.6 | 5.0 |
| *Mylopharyngodon piceus* | 2 | -21.2±1.6 | 7.3±0.3 | 18.7±1.3 | 122.4±41.6 | 2.7±0.1 |
| *Ctenopharyngodon idellus* | 3 | -20.8±0.9 | 6.6±0.4 | 19.4±0.8 | 150.3±23.6 | 2.4±0.2 |
| *Hypophthalmichthys molitrix* | 1 | -23.6 | 5.8 | 28.3 | 397.7 | 2.3 |
| *Hypophthalmichthys nobilis* | 6 | -24.3±1.5 | 10.5±1.2 | 19.5±3.4 | 176.8±110.5 | 4.1±0.5 |
| *Saurogobio dabryi* | 5 | -22.3±0.9 | 9.6±0.8 | 12.6±0.9 | 22.2±4.3 | 3.8±0.4 |
| *Coreius guichenoti* | 24 | -23.2±0.6 | 10.9±1 | 16.0±2.4 | 63.5±26.2 | 4.3±0.4 |
| *Coreius heterokon* | 6 | -27.1±1.3 | 9.8±0.8 | 27.2±2.1 | 283.5±69.3 | 4.0±0.4 |
| *Cyprinus carpio* | 3 | -22.0±1.9 | 6.8±1.5 | 20.6±11.5 | 549.4±814.9 | 2.6±0.5 |
| *Carassius auratus* | 10 | -21.7±1.1 | 7.9±1.2 | 15.0±1.8 | 105.5±36.9 | 3.0±0.4 |
| *Silurus asotus* | 10 | -22.8±1.0 | 11.3±0.8 | 24.5±8.7 | 175.4±289.7 | 3.8±0.3 |
| *Pseudobagrus albomarginatus* | 1 | -24.7 | 10.3 | 14.7 | 41.8 | 4.3 |
| *Pseudobagrus crassilabris* | 11 | -23.2±0.9 | 10.6±1.5 | 16.7±6.1 | 67.2±65.1 | 4.2±0.8 |
| *Tachysurus nitidus* | 4 | -24.1±1.2 | 9.8±1.2 | 9.9±0.6 | 12.3±2.0 | 3.7±0.5 |
| *Pseudobagrus vachelli* | 21 | -22.9±1.1 | 9.4±1.2 | 15.0±4.4 | 64.7±69.8 | 3.7±0.5 |
| **Downstream reach** |  |  |  |  |  |  |
| *Hemiculter leucisculus* | 8 | -24.1±0.9 | 8.8±0.8 | 11.7±3.2 | 18.6±11.6 | 2.9±0.4 |
| *Pseudolaubuca sinensis* | 2 | -24.9±1.7 | 8.2±1.3 | 11.9±0.1 | 23.2±0.3 | 2.8±0.6 |
| *Megalobrama amblycephala* | 1 | -24.2 | 9.9 | 14.1 | 74.3 | 3.0 |
| *Hemibarbus labeo* | 1 | -25.2 | 9.9 | 7.8 | 8.4 | 3.5 |
| *Hemibarbus maculatus* | 1 | -22.1 | 10.1 | 20.8 | 170.8 | 3.6 |
| *Saurogobio dabryi* | 8 | -22.5±0.5 | 8.7±0.7 | 13.0±1.2 | 29.7±9.4 | 2.9±0.3 |
| *Squalidus argentatus* | 1 | -22.1 | 11.1 | 8.8 | 11.8 | 4.0 |
| *Rhinogobio typus* | 4 | -22.5±0.4 | 10.2±0.3 | 21.8±2.1 | 101.3±25.3 | 3.6±0.1 |
| *Rhinogobio ventralis* | 6 | -25.0±1.3 | 9.6±1.2 | 16.4±2.2 | 81.4±36.6 | 3.3±0.5 |
| *Coreius guichenoti* | 6 | -15.0±18.3 | 8.2±0.9 | 20.1±1.8 | 141.3±30.8 | 2.6±0.4 |
| *Coreius heterokon* | 7 | -23.1±0.7 | 9.4±0.8 | 27.1±3.3 | 317.0±113.0 | 3.1±0.4 |
| *Cyprinus carpio* | 2 | -23.8±0.2 | 4.2±0.3 | 26.0±0.6 | 588.7±26.0 | 1.2±0.1 |
| *Carassius auratus* | 6 | -22.8±0.6 | 6.6±1.4 | 13.7±2.9 | 94.4±50.6 | 1.9±0.6 |
| *Garra pingi* | 1 | -22.2 | 7.7 | 10.5 | 19.4 | 2.5 |
| *Procypris rabaudi* | 1 | -23.4 | 7.9 | 13.6 | 48.6 | 2.6 |
| *Gobiobotia filifer* | 7 | -23.2±0.7 | 10.1±0.3 | 9.4±0.3 | 12.6±1.5 | 3.1±0.1 |
| *Xenophysogobio boulengeri* | 6 | -23.1±1.3 | 10.6±1.0 | 7.9±1.5 | 8.7±3.9 | 3.2±0.3 |
| *Leptobotia elongata* | 5 | -22.6±1.4 | 9.7±2.0 | 20.3±2.0 | 125.5±20.9 | 2.9±0.6 |
| *Leptobotia microphthalrna* | 3 | -24.0±1.1 | 9.0±1 | 12.8±1 | 34.1±8.9 | 3.1±0.4 |
| *Lepturichthys fimbriata* | 2 | -24.2±1.6 | 9.3±0.3 | 8.5±0.4 | 4.1±0.9 | 3.2±0.1 |
| *Jinshaia sinensis* | 3 | -24.2±0.3 | 8.1±0.7 | 9.2±0.9 | 13.6±3.9 | 2.3±0.2 |
| *Jinshaia abbreviata* | 2 | -25.4±0.5 | 8.0±0.5 | 7.8±0.7 | 10.3±4.6 | 2.6±0.2 |
| *Botia superciliaris* | 3 | -22.3±0.2 | 10.7±0.6 | 10±0.5 | 12.6±1.8 | 3.1±0.2 |
| *Silurus asotus* | 7 | -22.0±1.5 | 10.0±0.3 | 21.9±4.4 | 96.1±50.9 | 3.0±0.1 |
| *Glyptothorax sinense* | 1 | -25.9 | 10.7 | 6.5 | 5.5 | 3.3 |
| *Pseudobagrus crassilabris* | 13 | -23.2±1.4 | 9.9±0.9 | 13.2±4.2 | 32.6±38.3 | 3.3±0.4 |
| *Leiocassis longirostris* | 3 | -19.9±0.3 | 10.6±0.1 | 16.9±0.6 | 64.4±5.0 | 3.2±0.1 |
| *Tachysurus fulvidraco* | 3 | -23.2±1.2 | 9.4±0.5 | 12.1±0.8 | 35.3±5 | 3.2±0.2 |
| *Pseudobagrus vachelli* | 27 | -22.8±0.7 | 9.4±0.9 | 12.7±2.1 | 31.7±18 | 3.2±0.4 |


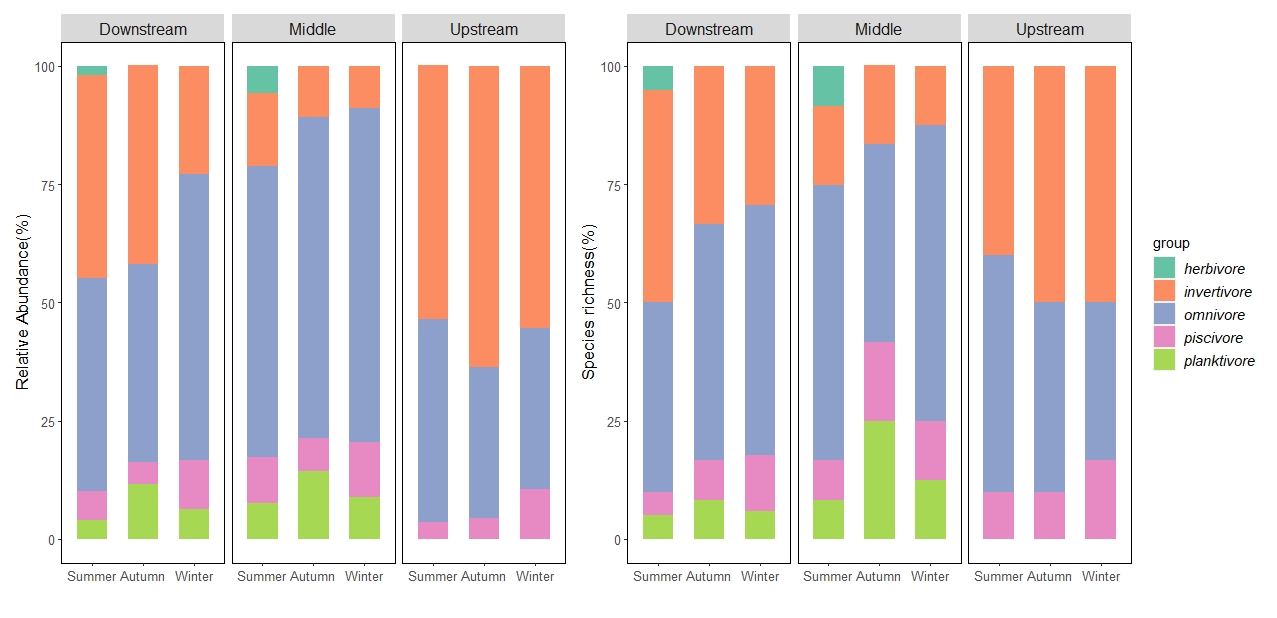
Figure s1 Proportions of the relative abundance and species richness of different feeding functional groups at each reach in the lower Jinsha River during summer, autumn, winter.
